# Neonatal Perforator Stroke: Timing, Risk Factors, and Neurological Outcome from a Single-Center Experience

**DOI:** 10.3390/neurolint17040059

**Published:** 2025-04-18

**Authors:** Andrea Calandrino, Gaia Cipresso, Marcella Battaglini, Samuele Caruggi, Irene Bonato, Paolo Massirio, Chiara Andreato, Francesco Vinci, Alessandro Parodi, Mariya Malova, Marta Bertamino, Elisabetta Amadori, Mariasavina Severino, Martina Resaz, Andrea Rossi, Pasquale Striano, Luca Antonio Ramenghi

**Affiliations:** 1 Department of Neuroscience, Rehabilitation, Ophthalmology, Genetics, Mother and Child Health, School of Medical and Pharmaceuticals, University of Genoa, 16132 Genoa, Italy; andrea.calandrino@edu.unige.it (A.C.); marcellabattaglini@gaslini.org (M.B.); samuelecaruggi@gaslini.org (S.C.); irenebonato@gaslini.org (I.B.); chiaraandreato@gaslini.org (C.A.); francescovinci@gaslini.org (F.V.); mariasavinaseverino@gaslini.org (M.S.); pstriano@unige.it (P.S.); lucaramenghi@gaslini.org (L.A.R.); 2 Neonatal Intensive Care Unit, Department of Maternal and Neonatal Health, IRCCS Istituto Giannina Gaslini, 16147 Genoa, Italy; paolomassirio@gaslini.org (P.M.); alessandroparodi@gaslini.org (A.P.); mariyamalova@gaslini.org (M.M.); 3 Physical Medicine and Rehabilitation Unit, Department of Pediatrics, IRCCS Istituto Giannina Gaslini, 16147 Genoa, Italy; martabertamino@gaslini.org; 4 Unit of Child Neuropsychiatry, Department of Pediatrics, IRCCS Istituto Giannina Gaslini, 16147 Genoa, Italy; elisabettaamadori@gaslini.org; 5 Pediatric Neuroradiology Unit, Department of Services, IRCCS Istituto Giannina Gaslini, 16147 Genoa, Italy; martinaresaz@gaslini.org (M.R.); andrearossi@gaslini.org (A.R.); 6 Department of Health Sciences, University of Genoa, 16132 Genoa, Italy; 7 Pediatric Neurology and Muscular Diseases Unit, IRCCS Istituto Giannina Gaslini, 16147 Genoa, Italy

**Keywords:** perforator stroke, neonatal arterial ischemic stroke, preterm, perinatal asphyxia

## Abstract

**Background and purpose:** Perforator stroke (PS) is a subtype of perinatal arterial ischemic stroke (PAIS), in which injuries occur in the territory of the perforator branches of the main cerebral arteries. This study aims to explore the incidence, timing, risk factors, and clinical presentation of PS in both preterm and full-term neonates. **Material and methods:** We retrospectively analyzed data about all the neonatal brain MRIs carried out in our hospital from March 2012 to March 2023. Criterium of inclusion was the radiologically confirmed diagnosis of perforator stroke involving one or more arterial districts. **Results:** A total of 1928 patients underwent brain MRIs during the period considered. PAIS was present in 50 patients, of which 19 had PS (38%). Among the patients with PS, nine were preterm babies (47%), and six suffered from perinatal asphyxia (31.5%). PS cUS diagnosis preceded MRI diagnosis in 88% of preterm babies. The mean age at cUS diagnosis was 20 ± 7 days. Preterm babies were often asymptomatic, whereas term babies showed neurological symptoms (mainly seizures). The outcome was favorable as long as PS was isolated. **Conclusions:** PS is surprisingly frequent among PAIS. It represents the most common form of PAIS in preterm babies and in babies suffering from birth asphyxia. Prenatal and perinatal factors suggesting a possible thromboembolic etiology leading to PAIS are rare in our population of preterm babies, in which the diagnosis was always preceded by negative cUS. These assumptions suggest a postnatal development of PS in premature babies more than a perinatal one.

## 1. Introduction

Perforator stroke (PS) is a subtype of perinatal arterial ischemic stroke (PAIS) in which vascular-related injuries occur in the territory of the perforator branches of the main cerebral arteries (anterior choroidal artery, anterior cerebral artery, middle cerebral artery, posterior cerebral artery, and posterior communicating artery), which supply the thalamus, striatum, posterior limb of the internal capsule (PLIC), centrum semiovale, and other structures [1]. The involvement of these important brain structures can cause potential impairment of neurodevelopment and cerebral palsy [2]. In particular, PS tends to affect deep brain regions, and its clinical impact can be severe, leading to long-term neurological sequelae such as cognitive deficits and motor impairments [2]. The adult counterpart of PS is a slightly rare event (reported to occur in around 0.32% to 5.1% of total arterial ischemic stroke cases), which is primarily associated with intracranial stenting and angioplasty, particularly in the basilar artery. It is influenced by procedural factors such as the proximity of the stented lesion to major perforators and plaque enhancement visible on high-resolution MRI [3,4].

Clinical symptoms of PAIS, and particularly of PS, may be difficult to recognize since they are extremely heterogeneous; the diagnosis of stroke is usually suspected in full-term infants presenting with seizures or apnea, while, on the contrary, preterm infants often do not display clinical symptoms; therefore, the diagnosis can be delayed or missed in this group [5,6,7]. As a result, PS is often underdiagnosed, and many cases are identified incidentally during routine neuroimaging. PS may present subtly, and the absence of immediate symptoms complicates its early detection [8].

Ultrasound is widely used in the NICU to investigate encephalopathic babies and as a screening tool for preterm neonates, and it can be a useful methodology for detecting PS [1]. While ultrasound is a valuable tool, it has limitations in detecting subtle signs of PS, and therefore, MRI remains crucial for a comprehensive diagnosis. However, very little is known about the risk factors, clinical presentation, and neuroimaging findings in PS; indeed, only one study in a large population of neonates with PS is available in the literature [8]. This study aims to explore the incidence, risk factors, and clinical presentation of PS in both preterm and full-term neonates; we report our experience with PS in a cohort of newborns of all gestational ages who underwent brain ultrasound (US) and MRI in our tertiary-level academic center for prematurity, neurological symptoms, prenatal malformation diagnosis, and birth asphyxia. Our goal is to provide a deeper understanding of the clinical and imaging features of PS to improve early detection and management strategies.

## 2. Materials and Methods

We retrospectively analyzed data about all the neonatal brain MRIs in our hospital from March 2012 to March 2023. Patients underwent brain MRI imaging for prematurity (postmenstrual age ≤ 32 weeks), perinatal asphyxia, prenatally diagnosed brain or spinal abnormalities, and neurological symptoms (including seizures, apnea, hypotonia, and hyporeactivity).

A radiologically confirmed diagnosis of PS involving one or more arterial districts was the inclusion criterion, while we excluded strokes with presumed or confirmed venous origin. For all these patients, we collected the prenatal and perinatal history, the presence of relevant anamnestic factors or relevant neurological manifestations, the results of histological placental examination, and the results of cUS and brain MRI. Fetal vascular malperfusion (FVM) was defined following the criteria provided by the Amsterdam Placental Workshop Group Consensus Statement; accordingly, thrombosis, segmental avascular villi, and villous stromal vascular karyorrhexis are placental histological findings consistent with FVM; other possible markers include avascular intramural fibrin deposition, stem vessel obliteration/fibromuscular sclerosis, and vascular ectasia [9]. Data about coagulation in our patients were collected. Prothrombotic screening at our center includes protein C and protein S levels, factor V and factor II mutation, and antithrombin III and factor VIII levels. The presence of central catheters was recorded as a risk factor for thromboembolism. In our center, we use umbilical venous catheters (UVCs) in patients with very low birth weight (VLBW) or critically ill patients to administer medications and parenteral nutrition. UVCs sized 3.5 ch or 5 ch (according to the patient’s weight) usually stay in place for 7 days at most, after which they can be replaced by epicutaneous-caval catheters (ECCs) sized 1 ch if needed. ECCs are removed or changed every 14 days. Patent ductus arteriosus (PDA) was defined as hemodynamically significant (hsPDA) when the ductal diameter was over 1.5 mm, the flow pattern was growing or pulsating, and when the patient showed clinical instability attributable to PDA. Patients with hsPDA were treated with paracetamol 15 mg/kg 4 times a day.

In our study, cUS screening was performed following a structured protocol. For neonates with GA less than 32 weeks and/or a birth weight under 1500 g, cUS was conducted within 24 h of birth, followed by screening on the third day. Subsequent cUS examinations were performed biweekly until the second week and weekly until discharge. Additional cUS was performed around term equivalent. For neonates with a GA of 32 weeks or greater and a birth weight of 1500 g or more, cUS was initially performed on the third day, followed by weekly screenings until discharge. In both groups, the frequency of cUS was increased if there were concerns about possible neurological abnormalities [10].

All the exams were performed using a GE, and MRI studies were acquired using a 3T Philips scanner (Ingenia Cx, Philips, Best, The Netherlands) and a 32-channel head-array coil. Standard imaging protocols included axial and coronal fast spin-echo (FSE) T2 weighted images, axial susceptibility-weighted imaging (SWI), isotropic fast-field echo gradient-recalled 3D-T1 weighted images, diffusion-weighted imaging (DWI), 3D pseudo-continuous arterial spin labeling (pCASL), and arterial and/or venous MR angiography with time-of-flight or phase-contrast techniques. Patients were fed before the MRI examination to achieve spontaneous sleep and were spontaneously breathing during the examination. Heart rate and oxygen saturation were constantly and non-invasively monitored.

Neurodevelopmental outcome was assessed at 2 years using the Griffiths Scales of Child Development, III edition tool.

Rough data are reported as absolute and relative frequencies and percentages; descriptive statistics for continuous variables are expressed as average and standard deviation or median and IQR. Fisher’s exact test was used to assess the presence of differences between the characteristics of the subcohorts. The significance threshold was set at 0.05, and results were obtained from two-tailed tests. Statistical analysis was conducted using SPSS for Windows, version 18 (SPSS Inc., Chicago, IL, USA).

## 3. Results

A total of 1928 newborns underwent an overall number of 2182 brain MRIs during the considered period. PAIS was present in 50 patients, among which 23 displayed neurological manifestations (46%), 15 (30%) were preterm babies who underwent MRI as a screening for internal protocol, and 12 were neonates with birth asphyxia (24%). PS was diagnosed in 19 newborns (38% of overall PAIS), of whom 3 manifested seizures, 1 hypoglycemia, 9 were preterm babies (8 VLBW—very low birth weight), and 6 suffered from perinatal asphyxia (16%, 5.5%, 47%, and 31.5% of all patients suffering PS, respectively). Among the asphyxiated newborns, metabolic acidosis at birth was present in two of three patients.

The diagnosis of PS was made by cUS (followed by a brain MRI for confirmation) or directly by MRI. Preterm babies were asymptomatic, and the diagnosis was incidental during the usual cUS screening or the brain MRI that we routinely perform at term-equivalent age (postmenstrual age of 40 weeks) for babies born before 32 gestational weeks. PS cUS diagnosis preceded MRI diagnosis in 88% of preterm babies. cUS diagnosis was made in 13 patients (68%), 8 of whom were preterms (61.5%), 2 had perinatal asphyxia (15%), and 3 displayed suspected seizures (23%). All the preterm patients had negative US findings during the first 2 weeks of life. The mean age at US diagnosis was 20 ± 7 days.

Table 1 shows PS patients’ characteristics and risk factors, divided into prenatal, perinatal, and postnatal groups. Placental histopathological data were available in nine cases (47%); in two patients (one preterm and one with asphyxia), there were signs of FVM. Thrombophilic screening was performed in seven patients, and showed alterations in one (increased IX factor levels).

All 19 patients had positive brain MRI; the mean time of MRI assessment was 38 weeks of postmenstrual age. In 10 patients (53%), MRI showed incidental findings besides PS: 4 patients had GMH or IVH, 2 showed alteration of the PLICs, 2 displayed a cerebellar hemorrhage, and 2 were shown to harbor other lesions (one dilatation of the subarachnoid spaces with incomplete Sylvian opercularization, and one with transverse sinus thrombosis). PS involved 23 perforator arterial branches, with a predominance of those of the middle cerebral artery (MCA) and the posterior cerebral artery (PCA); four patients reported two PSs simultaneously (Table 2).

In comparing the characteristics of preterm infants with PS and those with other types of PAIS (6 patients), the PS group shows a higher incidence of sepsis (77%) and patent ductus arteriosus (hsPDA) (66%), while asphyxia is more common in the PAIS-without-PS group (*p* < 0.05). The PS group also tends to have lower gestational age and birth weight compared to the PAIS-without-PS group, without reaching statistical significance. No other significant differences were reported.

Follow-up neuro-developmental data at 2 years of life were available for 13 patients: 2 were preterm babies with mild alteration of attention and signs of hyperactivity, of which 1 had two PSs simultaneously, and 2 were babies who suffered from perinatal asphyxia; at 2 years, they showed initial signs of hemiplegia, but they both had alterations of the PLIC other than PS, and one of them had two PSs simultaneously.

## 4. Discussion

PS is frequent in both term and preterm babies, and it represents 38% of all PAIS. Of interest, there are two subgroups of patients with a high incidence of PS: term babies suffering from birth asphyxia and exposed to hypothermia and VLBW babies undergoing MRI as a routine tool. In these two groups, PS represents the most common form of PAIS, and that, other than the possible lack of clinical symptoms, corroborates the importance of neuroradiological screening for these patients.

Regarding preterm babies, it is interesting to highlight the incidence of PS and PAIS in this category since several studies in the literature are focused on PAIS of term babies only without considering that preterms can also be at high risk of developing such lesions, although with a different symptomatology (or lack thereof) and probably a different etiology [11]. The incidence of PS in our preterm cohort is surprisingly high; PS appears to be the most frequent subtype of stroke, representing 60% of all PAIS (9 PS of 15 PAIS), confirming the data reported by Benders et al. in 2007 [12]. In the study of Ecury-Goossen, an incidence of just perforator stroke of 0.5% for infants born preterm was reported [8]. In the populations described by Raju et al. and Sorg et al., it was found that PAIS is more common in preterm infants than in full-term infants [13,14].

One possible explanation for the high incidence of PS in preterm infants could depend on the different anatomy between preterm and term babies: until 30–32 gestational week, a network of branches arising from the MCA connects the territories of the posterior and anterior cerebral artery, whereas after this age regression of the arterial system occurs, causing the formation of three separate territories. Thus, term babies have a higher risk of developing cortical ischemia, while in preterm babies, the extensive network of arterial connections between the three areas may provide sufficient blood to perfuse the cortex [15].

Furthermore, the distribution of deep cerebral perforators exhibits significant variability, with poor concordance rates among existing studies, except for the perforators of the posterior communicating artery and anterior choroidal artery [16]. Despite the assumption that the anatomy of cerebral perforators is consistent, studies reveal substantial variation in the distribution of different perforator groups [17]. This variability may help explain the frequent distribution patterns observed in PS and the lack of clearly identified, consistently strong risk factors. Moreover, studies conducted on histological specimens, functional MRI, and Doppler ultrasound have shown that perforator microcirculation in adults plays a crucial role in stabilizing the deep arterial circulation, allowing for the adjustment of the caliber of ostia based on flow velocities. In preterm infants, however, this mechanism is still immature, which we argue contributes to the increased frequency of PS in this population [18,19,20]. The lack of fully developed regulatory control over perforator vessel diameter may leave preterm neonates more vulnerable to ischemic events, further enhancing the incidence of PS. Additionally, the cerebral anatomy is still developing, this ongoing maturation could further contribute to the increased incidence of PS in this population, together with the fact that such babies are exposed to so many risk factors compared with term babies [14]. By the way, our group has already described significant differences occurring in the development of a profound venous system, also predisposing to IVH occurrence [21,22]. Similarly to our population, in the cohort reported by Ecury-Goosen et al. [8], three patients were diagnosed with PS after 28 days and after normal cUS. All of our preterm babies had normal ultrasounds before the diagnosis of PS, and this, plus the lack of malperfusion placental data (a well-described risk factor) [9], supports our hypothesis of a postnatal occurrence of PS in preterm babies. This could be related to the risk factors to which preterms are exposed during their stay in the NICU [12]; indeed, sepsis seems to be an important risk factor in preterm babies, with seven of nine of our preterm patients developing sepsis before the diagnosis of PS. The inflammation due to sepsis and the presence of central lines are important risk factors for developing symptomatic thromboembolic disease [23]; preterm infants in our cohort needed a central venous catheter for drugs and parenteral nutrition administration (mean age 38 days). Embolism has been frequently associated with placental thrombosis; histological placental data were available for nine patients, and signs of malperfusion were present in only two (one preterm), in agreement with Benders et al. finding no significant differences in placental histopathology between case infants and controls [12]. During the last year, a new protocol for central lines was started in our department, using heparin infusion in UVCs after the 5th day and in ECCs after the 12th day (when it is impossible to change the catheter) to avoid possible embolism. The results from this protocol are yet to be confirmed.

While PAIS is typically associated with well-established risk factors, primarily placental thromboembolism, which is absent in our study, PS appears to be more prevalent across both hemispheres without a clear lateral preference. This observation corroborates the assumption that the immaturity of the deep vascular system in preterm infants could play a significant role in the increased incidence of PS. Consistent with previous studies, perinatal stroke affects approximately 1 in 1600–4000 births, with a higher incidence observed in the left hemisphere [24]. This left-sided predominance may be attributed to systematic arterial asymmetries present in neonates, such as a larger left middle cerebral artery caliber and increased cortically directed blood flow [25]. However, in our study, PS does not show this typical lateral preference, further suggesting that the immature deep vascular system in preterm infants could contribute to the increased frequency of PS across both hemispheres.

Another possible explanation for the high incidence of PAIS in preterm babies, as suggested by Benders, could be the fact that these babies are routinely screened with cUS while neuroimaging in term babies is usually performed only when neurological symptoms occur [26]. cUS is confirmed to have a good sensitivity to diagnose PS (88% in preterm neonates) in agreement with Ecury-Goosen et al. [8], describing 80% of the diagnoses by cranial ultrasound, and with Abels et al. [27], who consider PS diagnosis to be feasible with brain US. Ecury—Goosen et al. [8] affirmed that PS was diagnosed in the first week of life in 60% of patients, and Steggerda et al. reported that these infarcts tend to become apparent beyond the first postnatal week [28]. In our cohort, the mean age at diagnosis was 20 days of life, which is why we prefer to refer to the first month of life (and not the first week) for the timing of diagnosis. Conversely, cUS was not useful for diagnosing PS in term babies, while only 1/8 VLBW with PS was missed at cUS. The low sensitivity of cUS in diagnosing PS in term babies may depend on the very subtle sign of PAIS in the first US scans as well as on the approach in asphyxiated infants who underwent cUS only at admission and MRI soon after hypothermia is concluded.

In our population, birth asphyxia was the most significant risk factor for developing PS, showing a clear perinatal etiology of PS as it represents 50% of all PAIS. On the other hand, of 140 babies with HIE, only 6 presented PS (4%). Ramaswamy et al. reported a similar incidence of PAIS in babies with asphyxia (4.8%), suggesting that neonatal encephalopathy, particularly when related to hypoxia-ischemia, should remain a risk factor for perinatal stroke [29]. In the literature, few studies are available about stroke in term babies with asphyxia; thus, the etiology remains unclear, but perinatal asphyxia is reported as a risk factor for PAIS in many studies [26,30,31]. Other recognized risk factors for PS are hypoglycemia, which was reported in one term baby in our cohort (5%), and twin pregnancy, reported in two cases (10%).

The clinical presentation represents a significant difference between our two populations of babies with PS; in fact, the diagnosis was incidental in all preterm babies who did not show symptoms suggestive of PAIS. Conversely, of the 10 term babies in our cohort, 6 were suffering from asphyxia, and all showed signs of encephalopathy, most frequently with hypotonia and hyporeactivity; 3 were term babies who seemed initially healthy and developed seizures. Seizures are a well-known symptom suggestive of arterial stroke; from our data of 17 patients with seizures and PAIS, 3 had PS (17%), so seizures may be considered a potential symptom suggesting PS. We observed seizures in three patients, all of whom were classified as having experienced birth asphyxia. Notably, one of these patients did not exhibit metabolic acidosis at birth. Based on the well-known involvement of deep basal ganglia lesions in birth asphyxia [30,32], we consider whether a PAIS exclusively affecting this territory unilaterally, such as a PS, may mimic hypoxic-ischemic encephalopathy symptoms. PS was asymptomatic in 80% of our patients. Ecury-Goosen et al. [8] reported 58% asymptomatic patients, and Abels et al. [27] described 15 asymptomatic patients in 24 patients with PS, as well.

**Figure 1:**
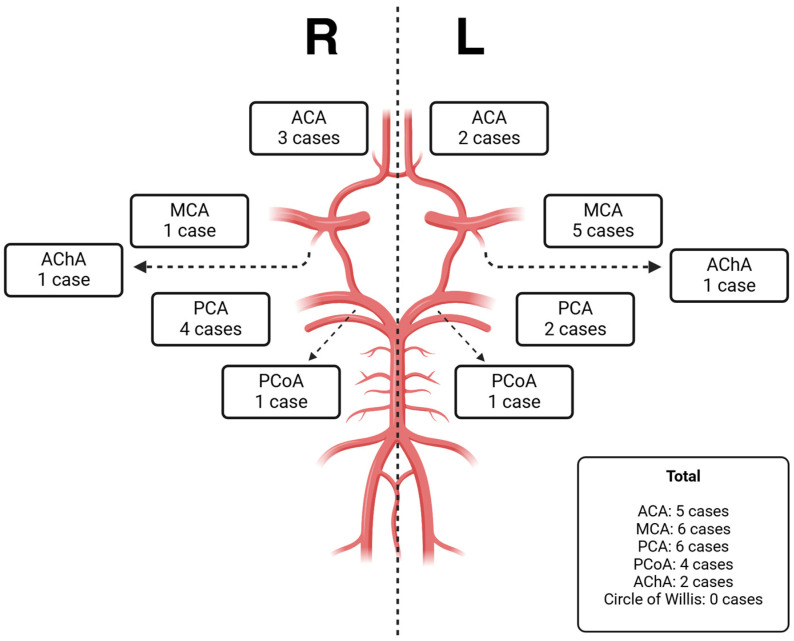
Location of perforator strokes. R: right; L: left; ACA: anterior cerebral artery; MCA: middle cerebral artery; PCA: posterior cerebral artery; PCoA: posterior communicating artery; AChA: anterior choroidal artery. Created in https://BioRender.com (accessed on 20 March 2025).

From our data, the most commonly affected territories in PS are those of the middle and posterior cerebral arteries, as shown in Figure 1. We agree with Ecury-Goosen et al. [8] in that there is no preferential hemisphere affected, in contrast with the described predominance of the left hemisphere for neonatal cortical arterial strokes [33].

PS seems to have a favorable outcome, even in patients with multiple PS without other lesions. Also, the outcome is different between the two populations of infants suffering from PS. Asphyxiated babies can show hemiplegia, especially if the PLICs are involved in the lesion. As previously reported by Ecury-Goosen et al. [34], PS has a favorable course if the PLICs are not involved. The importance of PLIC involvement was also reported by Abels et al. [27]. On the other hand, preterm babies usually do not manifest neurological impairment that can be connected with the PS.

The comparison between preterm infants with perforator stroke PS and those with other types of PAIS revealed key differences in risk factors. The PS group showed a higher incidence of sepsis and hsPDA, suggesting that postnatal factors may play a more significant role in the development of PS. Conversely, asphyxia was more common in the term infants, highlighting the abovementioned differences in terms of anatomical background in the pathogenesis of PS [35].

Although the PS group tended to have lower gestational age and birth weight compared to the PAIS-without-PS group, this did not reach statistical significance. Overall, these results support the notion that PS and non-PS PAIS may have distinct risk profiles, with PS being more closely linked to postnatal factors like placental malperfusion [9], while non-PS PAIS appears more strongly associated with perinatal events like asphyxia.

In conclusion, while PS has been studied primarily in full-term infants, our research highlights the unexpectedly high incidence of PS in preterm neonates, with a significant focus on VLBW infants. We hope this contributes to filling a gap in the current understanding of PS, especially considering the unique vulnerability of VLBW preterm babies to this subtype of PAIS. Our findings challenge previous assumptions about PS and emphasize the importance of early detection in these high-risk populations, where PS may often go undiagnosed due to its asymptomatic presentation and postnatal onset [11].

As for future direction, since the etiology of perforator stroke remains uncertain, it may be useful to investigate possible causes of cerebrovascular diseases in the elderly, considering the numerous characteristics shared by frail individuals at the extremes of life (elderly and neonates). A possible hypothesis could be explored within the group of cerebral small vessel diseases (cSVDs), which, as described in the literature, are associated with stroke in adults [36]. 7T-MRI has been used to assess perforating artery stiffness and altered flow velocities in adult patients with cSVDs [37,38]. Similar studies in neonates could shed light on whether this kind of alteration is present in preterm and term babies and whether it might be associated with neonatal perforator stroke. Of course, safety issues for 7T in small infants must be considered.

## 5. Conclusions

We showed a surprisingly high incidence of PS among PAIS. Indeed, PS represented the most common form of PAIS in preterm babies and in babies suffering from birth asphyxia in our series. We remain uncertain about several of the steps linking the etiology of these lesions and their development in these two categories of infants. PS is often diagnosed incidentally and may occur after multiple normal brain sonographic screenings. PS is usually asymptomatic and has a favorable outcome when isolated. Previously described risk factors for PS, such as asphyxia and sepsis, are confirmed in our population. Conversely, prenatal and perinatal factors triggering a possible thromboembolic etiology leading to PAIS were rare in our population of preterm babies. These observations suggest that PS in preterm neonates likely develops postnatally rather than perinatally. The term PAIS may, therefore, be inappropriate for describing these strokes in premature infants, as their onset typically occurs after birth. PS, in particular, is frequently associated with postnatal risk factors such as sepsis, the use of central venous catheters, and other NICU-related conditions rather than events occurring in utero or around the time of delivery.

Given this temporal distinction, we propose adopting (preterm) neonatal arterial ischemic stroke (pNAIS, or NAIS) to better reflect the postnatal nature of these ischemic events in preterm neonates. This change would more accurately align the terminology with the clinical reality that many neonatal strokes in this population occur after birth. We believe that using (p)NAIS would enhance communication in clinical practice and research, facilitating a better understanding of these strokes, improving early detection, and promoting more effective management strategies, particularly in preterm infants. Additionally, this shift would foster more targeted research into the unique causes, outcomes, and treatments for neonatal strokes in this population.

As the classical perinatal stroke etiology, such as placental embolism, is excluded in these cases, it becomes crucial to explore other potential underlying mechanisms. We propose that future studies investigate the role of thrombophilia in preterm infants with PS, as this may provide a better understanding of the pathophysiology of stroke in this vulnerable population, which could also help explain the observed association with IVH [39].

According to existing recommendations, thrombophilia was not assessed in our population, which may limit our ability to fully explore its potential role in the development of PS in preterm infants [40].

The current study’s limitations are its retrospective and monocentric nature. However, we believe this report adds valuable information about PS, an entity still poorly described in the literature. More studies involving different centers are awaited to shed light on neonatal PS’s etiopathogenesis, presentation, and outcome.

## Figures and Tables

**Table 1:** PS population characteristics and risk factors.

Patients’ Characteristics	Preterm Babies (9)	Term Babies (10)
Sex	5F (55%), 4M (45%)	8M (80%), 2F (20%)
Gestational age (median)	27 GW	38 GW
Birth weight (mean ± SD)	1060 ± 430 g	3240 ± 520 g
BW percentile (median; 25th–75th pc)	60; 32–86.5	52; 29.75–77
Body length percentile (median; 25th–75th pc)	46; 34.5–65	50; 34.5–65.5
Cranial circumference percentile (median; 25th–75th pc)	44; 22.5–62	40; 22.75–58.25
SGA (N;%)	1 (11%)	1 (10%)
IUGR (N;%)	2 (22%)	0
Twin gestation (N;%)	2 (22%)	0
APGAR 1′ (median)	6.5	5
APGAR 5′ (median)	8	7
CS (N;%)	6 (66%)	5 (50%)
**Risk factors**		
**Prenatal**		
Placental malperfusion	1 (11%)	1 (10%)
Placental abruption	3 (33%)	1 (10%)
Pre-eclampsia	1 (11%)	2 (20%)
**Perinatal**		
Asphyxia	0	6 (60%)
Urgent CS	5 of 6 CS (83%)	5 of 5 CS (100%)
**Postnatal**		
hsPDA	6 (66%)	0
Central venous catheter	9 (100%)	10 (100%)
Altered coagulation (tot 7)	1 (11%)	0
Sepsis	7 (77%)	0

SGA: small for gestational age; IUGR: intrauterine growth restriction; CS: Cesarean section; hsPDA: hemodynamically significant patent ductus arteriosus.

**Table 2:** Reports the neuroradiological features and neurodevelopmental follow-up for all patients.

	GA	MRI Indication	PS Number	Vessel Interested	MRI Incidental Findings	Neurological Impairment
1	27	Prematurity	1	PCA L	IVH	n.a.
2	29	Prematurity	1	pcOa R	IVH	0
3	25	Prematurity	2	PCOA + ACA R	0	0
4	40	HIE	2	PCOA + ACA R	0	0
5	32	Prematurity	1	ACA L	0	Attention deficit and hyperactivity
6	39	Hypoglycemia	1	PCA R	0	0
7	33	Prematurity	1	ACA R	Dilatation of the periencefalic spaces with incomplete Sylvian opercolarization	0
8	37	HIE	1	MCA L	PLIC’s alteration	Hemiplegia
9	36	HIE	1	PCA L	0	0
10	40	HIE	2	AChA + PCoA L	PLIC’s alteration	Hemiplegia
11	25	Prematurity	2	PCA + AChA R	IVH	Attention deficit and hyperactivity
12	25	Prematurity	1	MCA L	IVH	0
13	29	Prematurity	1	PCA R	0	0
14	36	HIE	1	MCA R	0	n.a.
15	39	Seizures	1	MCA L	Transverse sinus thrombosis	n.a.
16	36	Seizures	1	MCA L	0	n.a.
17	37	Seizures	1	MCA L	0	0
18	40	HIE	1	ACA L	CBH	n.a.
19	23	Prematurity	1	PCA L	CBH	n.a.

GA: gestational age; HIE: hypoxic-ischemic encephalopathy; ACA: anterior cerebral artery; MCA: middle cerebral artery; PCA: posterior cerebral artery; PCoA: posterior communicating artery; AChA: anterior choroidal artery; L: left; R: right; IVH: intraventricular hemorrhage; CBH: cerebellar hemorrhage; PLIC: posterior limb of the internal capsule; n.a.: not available.

## Data Availability

The original contributions presented in the study are included in the article, further inquiries can be directed to the corresponding author/s.

## Abbreviations

The following abbreviations are used in this manuscript:

PS	Perforator stroke
PAIS	Perinatal arterial ischemic stroke
cUS	Cranial ultrasound
MRI	Magnetic Resonance Imaging
NICU	Neonatal Intensive Care Unit
VLBW	Very low birth weight
PDA	Patent ductus arteriosus
hsPDA	Hemodynamically significant patent ductus arteriosus
UVC	Umbilical venous catheter
ECC	Epicutaneous-caval catheter
FVM	Fetal vascular malperfusion
FSE	Fast spin-echo
SWI	Susceptibility-weighted imaging
DWI	Diffusion-weighted imaging
pCALS	Pseudo-continuous arterial spin labeling
SGA	Small for gestational age
IUGR	Intrauterine growth restriction
CS	Cesarean section
IVH	Intraventricular hemorrhage
PCA	Posterior cerebral artery
MCA	Middle cerebral artery
ACA	Anterior cerebral artery
PCoA	Posterior communicating artery
AChA	Anterior choroidal artery
PLIC	Posterior limb of the internal capsule
HIE	Hypoxic-ischemic encephalopathy
CBH	Cerebellar hemorrhage
n.a.	Not available
IQR	Interquartile range
(p)NAIS	(Preterm) neonatal arterial ischemic stroke
